# Balancing expectations amidst limitations: the dynamics of food decision-making in rural Kerala

**DOI:** 10.1186/s12889-015-1880-5

**Published:** 2015-07-12

**Authors:** Meena Daivadanam, Rolf Wahlström, K.R. Thankappan, T.K. Sundari Ravindran

**Affiliations:** Achutha Menon Centre for Health Science Studies, Sree Chitra Tirunal Institute for Medical Sciences and Technology, Thiruvananthapuram, 695011 India; Department of Food, Nutrition and Dietetics, Uppsala University, Box 560, SE-751 22, Uppsala, Sweden; Department of Public Health Sciences (Global Health), Tomtebodavägen 18A, Karolinska Institutet, 171 77 Stockholm, Sweden; Family Medicine and Preventive Medicine, Department of Public Health and Caring Sciences, Uppsala University, Uppsala, Sweden

**Keywords:** Food decision-making, Household, Non-communicable diseases, Behavioural intervention, Focus groups, Content analysis, Kerala, India

## Abstract

**Background:**

Food decision-making is a complex process and varies according to the setting, based on cultural and contextual factors. The study aimed to understand the process of food decision-making in households in rural Kerala, India, to inform the design of a dietary behaviour change intervention.

**Methods:**

Three focus group discussions (FGDs) and 17 individual interviews were conducted from September 2010 to January 2011 among 13 men and 40 women, between 23 and 75 years of age. An interview guide facilitated the process to understand: 1) food choices and decision-making in households, with particular reference to access; and 2) beliefs about foods, particularly fruits, vegetables, salt, sugar and oil. The interviews and FGDs were transcribed verbatim and analysed using qualitative content analysis.

**Results:**

The analysis revealed one main theme: ‘Balancing expectations amidst limitations’ with two sub-themes: ‘Counting and meeting the costs’; and ‘Finding the balance’. Food decisions were made at the household level, with money, time and effort costs weighed against the benefits, estimated in terms of household needs, satisfaction and expectations. The most crucial decisional point was affordability in terms of money costs, followed by food preferences of husband and children. Health and the risk of acquiring chronic diseases was not a major consideration in the decision-making process. Foods perceived as essential for children were purchased irrespective of cost, reportedly owing to the influence of food advertisements. The role of the woman as the homemaker has gendered implications, as the women disproportionately bore the burden of balancing the needs and expectations of all the household members within the available means.

**Conclusions:**

The food decision-making process occurred at household level, and within the household, by the preferences of spouse and children, and cost considerations. The socio-economic status of households was identified as limiting their ability to manoeuvre this fine balance. The study has important policy implications in terms of the need to raise public awareness of the strong link between diet and chronic non-communicable diseases.

**Electronic supplementary material:**

The online version of this article (doi:10.1186/s12889-015-1880-5) contains supplementary material, which is available to authorized users.

## Background

Unhealthy diet is one of the major risk factors for non-communicable diseases (NCDs), which include cardiovascular and respiratory diseases, mental disorders, diabetes and cancer. NCDs are the leading cause of death and disability in India [1, 2]. According to the million-death study, cardiovascular diseases are the leading cause of death (42 %) in all regions of India, and it is highest in the South Indian state of Kerala [3]. Kerala also has the highest prevalence of diabetes and NCD risk factors [4, 5].

Among all Indian states, Kerala is the most advanced in terms of epidemiologic and demographic transition [5]. The state has also seen a similar shift as the rest of India and the world in diet and physical activity patterns, referred to as Nutrition Transition [6, 7]. Dietary habits in Kerala vary considerably from region to region and among different castes and religions [8, 9]. However, there is a documented increase all over Kerala in consumption of animal source foods and dairy products [10–12]; and very low per capita consumption of fruits and vegetables (FV), excluding roots and tubers [12]. Findings from a dietary survey showed that 70 % of fat energy was derived from saturated fats at every level of energy consumption [13]. The fat profile of the Kerala diet is heavily loaded towards saturated fats, by the regular use of fresh, grated coconut, and, to a lesser extent, coconut oil [13, 14]. Similarly, Kerala had no ‘respectable’ restaurants about two decades ago, only a few teashops and ‘meals hotels’ [15]. Today, the rapid growth of restaurants and eateries in Kerala depicts both a change in attitude towards eating out influenced by media images of ‘good living’; and rapid investments due to the entry of big players into the market [15].

There is strong scientific support to link food consumption to three of the NCDs currently in focus, i.e., cardiovascular diseases, diabetes and cancer, which forms the evidence base for most NCD prevention efforts involving diet [16]. However, convincing evidence linking diet and NCDs exists only for a few food groups and dietary components. First, low FV intake is among the top 10 risk factors contributing to attributable mortality [17]. The WHO guidelines recommend at least 400 g of FV intake per day [18]. Second, a high dietary salt intake is significantly associated with high blood pressure [19]; and high risk of stroke and cardiovascular disease [20]. The major source of dietary salt is processed foods in most high-income countries; and home-cooked foods in most low-and middle-income countries [21]. International health bodies like the WHO and World Heart Federation among others, recommend a daily intake less than 5–6 g salt [19, 22]. Third, the most consistent association between sugars and an increased risk of obesity has been established in relation to two sources of intake: high consumption of sugar-sweetened beverages, in both adults and children [16, 23, 24]; and free sugars as defined by the WHO-FAO expert consultations and the WHO scientific updates (“all monosaccharides and disaccharides added to foods by the manufacturer, cook, or consumer, plus sugars naturally present in honey, syrups, and fruit juices” [16], p 109). Otherwise, the evidence linking sugar consumption to NCDs has been inconsistent and controversial and sugars are also defined in multiple ways, which adds to the confusion. Current recommendations suggest restricting intake of free sugars to less than 10 % of total energy intake [16]. Fourth, there is convincing evidence of significant coronary risk reduction by replacing saturated fatty acids with poly-unsaturated fatty acids, particularly linoleic acid [16, 25]. The role of saturated fatty acids, particularly myristic and palmitic acids (meat products) in raising the total cholesterol and low-density lipoprotein; and the double atherogenic effect of trans-fatty acids (processed foods) in elevating the low-density lipoprotein and reducing high-density lipoprotein at the same time has also been consistently established [16, 25]. The current dietary recommendations suggest a total fat intake between 15 and 30 % of total energy intake, with consumption of saturated fats restricted to less than 10 % and trans-fats to less than one percent of total energy [16]. As with the case of sugar, the evidence against different types of fats, in terms of cardiovascular disease risk is controversial and inconsistent [16].

Although scientific evidence convincingly associates dietary content to health effects, we know that food decision-making is a complex process and involves more than taking into account the nutritive value of the foods. It also varies according to the setting, based on cultural and contextual factors [26]. Decision-making related to food has been studied predominantly from marketing, sociological or economic perspectives [27–31], not from the health perspective [32–34]. The high prevalence of chronic non-communicable diseases (NCD) and their risk factors in rural Kerala, India [5], calls for more in-depth knowledge on decision-making for food-related matters [35] in order to gain a deeper understanding of the process. A few studies from India have looked at decision-making in households from a consumer perspective with food as one of the commodities [31]; and at bargaining power and its implications in terms of gender-power relations [27, 30].

Food-related decisions have more or less universally been regarded as a woman’s domain [28, 31], although other members influence this decision to varying degrees through their stated or unstated preferences [8, 32, 36]. Family members’ different bargaining power [27] seems to be a major determinant in the process. In more egalitarian societies, bargaining power may be distributed more evenly or could be negotiated based on circumstances and context [37]. In hierarchical societies, however, this may rest disproportionately with one or two individuals within a household [37]. A person’s bargaining power within his or her household is determined by his or her “ability to physically survive outside the family” [27], p. 9, which is traditionally measured in terms of income, education and occupation [30]. These three “traditional determinants of status in social stratification” [30], p. 625 also stratify members in a household, and explains the lower status and low bargaining power that women have in most traditional patriarchal societies [27].

There have been many attempts to understand food choices and the food decision-making process [26, 33, 38, 39]. The main body of work related to food decision-making has come from Cornell University, through the two Grounded Theory models: the Food Choice Process Model [36, 40]; and the Food Decision-Making Framework [32, 34]. Gillespie et al. arrived at seven broad propositions that characterize family food decisions to develop their framework [34]. The Food Choice Process Model on the other hand looks more at the individual, and how their life-course experiences shape their personal experience with food and thereby, influence their food choices [36].

In spite of the differing nature of the societies, where these studies have been carried out [37], there are certain common factors. In consumer research, food is a commodity that is consumed jointly by members of a household; and greater family influence has been hypothesized for such commodities [28]. Hence, irrespective of culture, food choices and food decision-making involve a greater collective component.

To inform the design of a community-based dietary behavioural intervention [41], we required more context-specific information and understanding of this process. The aim of this study was thus to explore the process of food decision-making in relation to chronic non-communicable diseases within rural households in Kerala.

## Methods

### Study setting

This was a qualitative study using focus group discussions (FGDs) and individual interviews; and is described here based on RATS guidelines for reporting qualitative studies [42]. It was conducted as part of the formative research for a community-based dietary behaviour change intervention for prevention of chronic non-communicable diseases in rural Kerala [41]. Thiruvananthapuram district, with a population of about 3.3 million inhabitants, is the home of the public health institution where three of the co-authors are based. Kerala follows a de-centralised system of administration at the state, district, taluk and block-panchayat levels, with the latter being further de-centralised to rural (*grama panchayat*) and urban (municipality) administrative units [43]. Kerala holds a unique position in India because of its high female literacy and the highest score for the Gender Development Index [44]. *Chirayinkeezhu* taluk is one of four taluks of Thiruvananthapuram district with a population of 550 thousand (about 130 thousand households). It is divided into four block panchayats, which in turn consists of 22 *grama panchayats* and 2 municipality areas. The study was conducted in the rural areas in two of the *grama panchayats*, one of which was coastal and the other non-coastal. Kerala has a well-developed and functioning women’s self-help group network called the *Kudumbasree*, which is organised in the form of neighbourhood groups or *ayalkootams*. We identified different socio-economic areas through the *Kudumbasree* registers, and the individual households were then sampled purposively from the identified localities.

### Participants

The participants were men and women aged between 23 and 75 years, and of different religions and socio-economic strata (SES). While the 17 participants in the individual interviews belonged to different SES (low-3; middle-8; high-6); participants in FGD 1 (7 women) and 2 (9 women) were predominantly from low and low-middle SES. The study focused primarily on food decision-makers in rural households in Kerala. Since women are predominantly involved in food procurement and preparation, the first two focus groups and five of the individual interviews focused on female heads of the households. From the sixth interview, the female head of household was informed that she could bring any other household member who was also involved in decision-making related to food matters in their household to the interview. The third FGD was organized with twelve men from both low-middle and high-middle SES. There was no overlap among participants between the FGDs and the individual interviews.

### Data collection procedure

The three FGDs and 17 individual interviews were arranged through community volunteers, who resided in the area and interacted with the people on a regular basis. The volunteers approached residents by locality and requested time for the respective events, and all agreed. All interviews were conducted in the residences of the participants, except one, which was conducted in a neighbour’s house. FGDs 1 and 2 were conducted in a local school, while FGD 3 was conducted in the residence of one of the participants in the coastal village. Four women joined their spouses in this focus group and their contributions have been included in the analysis. Each FGD lasted for about 1.5 hours, while the interviews varied between 45 minutes to 1.5 hours. No further interviews were conducted once saturation was achieved. All interviews were conducted between September 2010 and January 2011 in the local language, Malayalam and recorded using digital voice recorders. The interviews and FGDs were transcribed verbatim in Malayalam. Member checks were not done, but in a few cases clarifications were sought directly from the concerned participants.

Observations at market places and provision stores were also undertaken to understand the dynamics involved in the purchase of fruits and vegetables.

### Interview and FGD guide

Both the interviews and FGDs were conducted based on an interview guide (Additional file 1) to explore: 1) food choices and decision-making in households, with particular reference to access; 2) beliefs about foods, particularly fruits and vegetables (sections I-IV, Additional file 1); 3) feasibility and acceptability of a proposed dietary behaviour change intervention; and 4) the kind of strategies that may be practical at community and household level (section V, Additional file 1). An informal assessment (section VI, Additional file 1) of household consumption or procurement of salt, sugar, oil and coconut was also done. For the present study as well as the intervention trial, we specifically focused on food components added by the cook at home, which would make it possible to separately measure the components of the consumed foods. As the purpose was to understand the background and factors needed to develop a practical and context-specific intervention, the interviews and focus groups concentrated on five dietary components: fruits, vegetables, salt, sugar and oil. These five dietary components were chosen based on convincing evidence linking these to NCDs [16]. Most of the snacks and beverages consumed in the study areas were locally produced and they were addressed to a small extent but mainly as discreet items on their own, rather than as additional sources of salt, sugar or oil. As it was difficult to standardize their contents without separate food component analysis, we decided to focus primarily on the components where any change that took place as a consequence of the intervention could be accurately measured. So, the decision-making in households also looked at food in relation to these five components and specifically in relation to non-communicable diseases. This was conveyed to the participants both in the written information sheet as well as during the introduction to the interviews. Hence, the analysis has been carried out with the basic understanding that the responses of all the participants were predominantly focused on these five components unless specifically asked otherwise.

### The research team

The research team included the principal investigator (MD), an assistant, one Swedish (RW) and two Indian (KRT, TKSR) public health scientists. The first author (MD), who was also the interviewer and moderator is a medical practitioner with public health training and provided an insider perspective. An assistant, who was the note-taker, accompanied her for the FGDs. TKSR and RW are well experienced in qualitative research and looked at the data with insider and outsider perspectives respectively. KRT being a medical and public health practitioner in the state also provided an insider perspective.

### Analysis

Data related to section I to IV of the interview guide was analysed for this study using manifest and latent content analysis [45] in the local language, Malayalam. Direct translation of the transcripts was done to allow crosschecking by RW and TKSR, for whom Malayalam was not their primary language. MD conducted the primary analysis; codes and themes were crosschecked by RW and confirmed by TKSR. Meaning units were identified from the data and coded in an Excel spreadsheet. Codes were coalesced to identify manifest categories and emerging sub-themes and latent themes. Any differences of opinion were discussed before themes were finalised.

The initial manifest content part of the analysis dealt only with ‘visible and obvious components’ of the text. Then we continued with a latent content analysis to interpret the underlying meaning of the text [45]. Meaning units were initially identified from the text, condensed and coded. Once the whole data was coded, codes were coalesced to form sub-categories and then abstracted further to form categories, sub-themes and the main theme (Example shown in Additional file 2). Since it was necessary to abstract beyond the data to understand the underlying meanings, we used at least five levels of abstraction from the codes. Graneheim and Lundman describe a theme as “a thread of an underlying meaning through the condensed meaning units, codes or categories on an interpretative level”, and thus answering the question ‘How?’ [45], p 107. Through our analysis, we have attempted to understand how food decisions were made in rural households in Kerala.

The complete data which included the sections I to IV were also analysed separately using modified framework analysis in another study by the authors to build a conceptual model that could facilitate the development of strategies for a household-level dietary behaviour change intervention [46].

### Ethical considerations

This study was conducted according to the guidelines laid down by the Indian Council of Medical Research. The Institutional Ethics Committee of Sree Chitra Tirunal Institute for Medical Sciences and Technology, Thiruvananthapuram, Kerala, India approved all the procedures involving the study participants as well as the interview and FGD guidelines. Participants were recruited only after recording verbal informed consent.

## Results

The demographic characteristics of the participants in the FGDs and individual interviews are described in Table 1. Only one of the women had formal employment, while many of the others were informally engaged in fish vending or shop keeping. Four women opted to be interviewed together with another household member, including: the husband (main interviewee: wife); widowed mother staying with married daughter (main interviewee) and her family; widowed mother-in-law staying in her son’s house (main interviewee: daughter-in-law) and daughter who was staying with her family in her mother’s (main interviewee) house.

**Table 1:** Characteristics of participants in focus groups and individual interviews

Type	Participants (n)	Age range	Median age	Gender
Focus group 1	7	40-66	57	M-0; F-7
Focus group 2	9	26-67	41	M-0; F-9
Focus group 3	16	26-73	59	M-12; F-4
13 interviews-individuals	13	23-75	44	M-0; F-13
4 interviews-Pairs	8	24-63	47	M-1; F-7

M = male, F = female

The staple foods of this area are rice or rice-based foods, legumes or pulses, and fish, particularly along the coastal area where fishing is the major occupation. This predominantly rural area has both low and middle SES areas, and here the consumption of branded beverages, meat based products and processed foods is not a regular feature as in the more affluent or urban areas. Coconut oil is the predominant cooking oil used and coconut scraping is an ingredient in every meal. The term fruit is almost synonymous for different types of bananas. There are some locally available fruits such as guavas and gooseberries, which are available throughout the year, and others such as jackfruit and mango, which are seasonal. The most common vegetable curry is called ‘*sambar*’, which is a mixed vegetable preparation. Though consumption of vegetables is low in the state, most households have at least one vegetarian dish or curry daily, which in poorer households may contain only coconut scrapings.

### How are food-related decisions made?

**Fig. 1:**
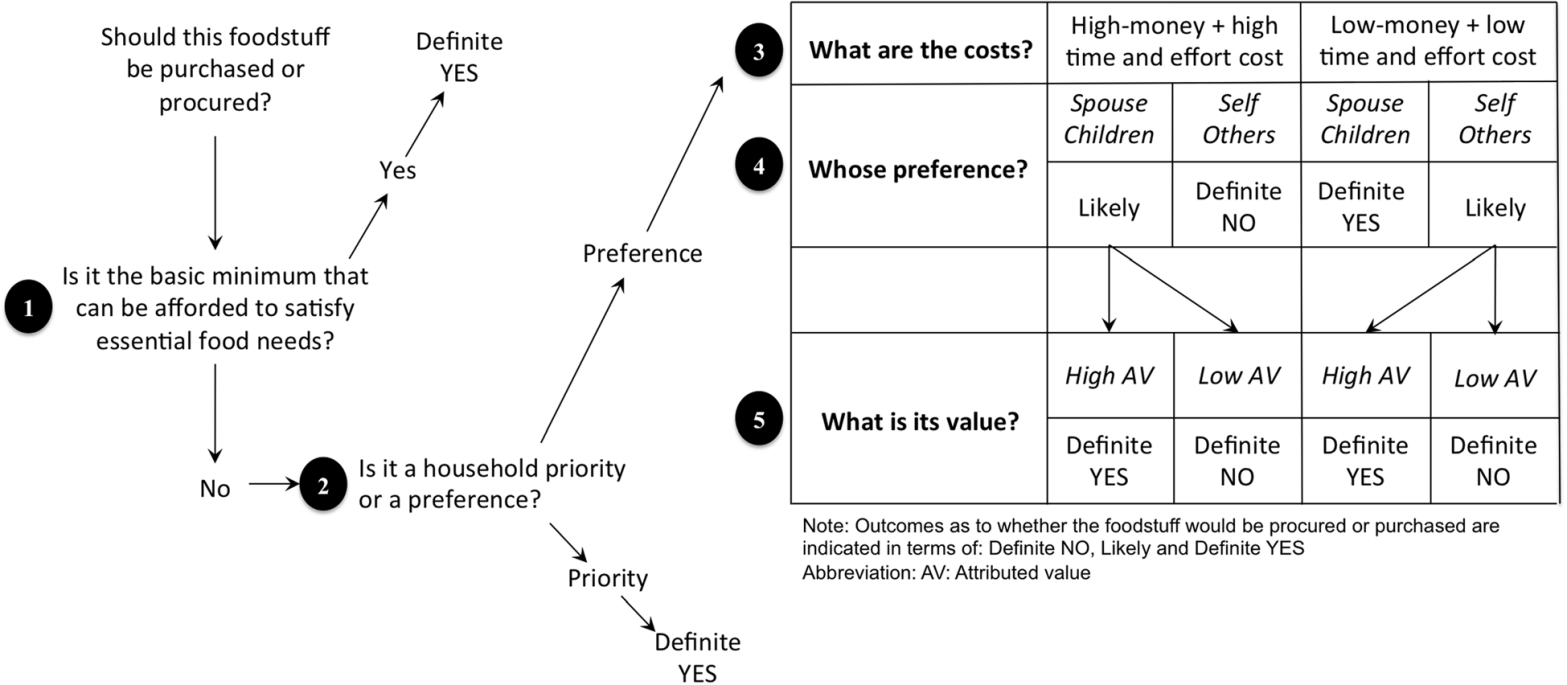
Decision tree for the process of prioritization and food decision-making in households. This decision tree is constructed based on our findings, focusing purchase or procurement of five dietary components: fruits, vegetables, salt, sugar and oil. It describes the prioritization process in terms of five key questions to be considered in that order. *Priority* was considered as essential based on the disease status or age of household members, particularly spouse and young children. *Preference* was based on habit or taste of the more influential members of the household, specifically spouse and children. We have described only two cost options: ‘high money + high time and effort cost’ and ‘low money + low time and effort cost’, as these can be clearly linked to the data. Note that the outcomes described in the table are combinations of preceding steps, e.g., if a food item comes with high costs, the household is likely to procure it if it is the preference of spouse or children; and they will definitely procure it if the food also has a high attributed value, but not if it has a low attributed value

The results of the qualitative manifest and latent content analysis are shown in Table 2. The analysis revealed two sub-themes ‘counting and meeting the costs’ and ‘finding the balance’ and one main theme ‘balancing expectations amidst limitations’. The following section first describes the sub-themes and categories, while the main theme is summarized at the end of this description. Each of the categories are also characterized in terms of a main question (shown within brackets), which is used to construct the decision-tree (Fig. 1). All quotations are in italics and any text within the quote enclosed by square brackets have been inserted by the authors.

**Table 2:** Results of the qualitative content analysis on food decision-making process at the household level

Categories	Sub-themes	Theme
1. Monetary and other costs	Counting and meeting the costs	Balancing expectations amidst limitations
*What are the costs involved?*		
2. Living within our means		
*Can we afford basic needs?*		
3. Meeting household needs	Finding the balance	
*Is it a household priority?*		
4. Maximizing household satisfaction		
*What is its value?*		
5. Matching roles and expectations		
*Whose preference is more important?*

### Sub-theme 1: Counting and meeting the costs

The first sub-theme primarily relates to different types of costs involved in the procurement and preparation of foods in rural households and how households meet these costs.

*Category 1: Monetary and other costs (What are the costs involved?)*

The cost of procuring foods included the direct cost in terms of the market price of the actual commodity and its transportation; and indirect costs in terms of time and effort spent on the exercise.

Money costs were found to be the most important and recurring theme through all interviews and transcripts, and mainly comprised the various expenses involved in procuring the food, including travel. Here, money was considered in actual monetary terms or in-kind, particularly related to the produce from land-holdings and neighbourhoods in the form of wild or locally produced varieties of fruits and vegetables. In most rural areas in the study district where land was fertile, this meant a source of cheap and readily available foods, particularly fruits like papaya and jackfruit, and vegetables like greens and drumsticks, either from one’s own yard or one’s neighbour’s: “*In our area, we have mostly used drumstick trees for fencing, so during rains, they grow. We get drumstick leaves and sometimes the fruits also for different dishes*”. (FGD 1; Female; 40 years).

On the other hand, when landholdings were too small; or infertile with unfavourable terrain and conditions, like coastal areas with salty sand, or water-logged areas, then it was felt not worthwhile to grow fruits and vegetables. In such circumstances, the monetary costs of procurement became an important consideration. When the *chantha* (local market) was close-by, this was mitigated to some extent. These *chanthas* usually functioned in most localities. There are large *chanthas* in the towns and cities for fruits and vegetables, fish and meat. Most communities however, had small wayside *chanthas* on specific weekdays, mostly for vegetables and fish, where one can get locally grown produce as well. They also explained that it was more economical to buy a cut vegetable pack, rather than buy separate vegetables for different dishes. Local markets catered to this demand by keeping ready-made packs for fixed prices (based on current price) or putting together such a pack right in front of you. All one had to do was ask for a 25-or 50-rupee packs, depending on one’s budget and the available pack sizes.

Money costs were however not the only consideration as time and efforts costs were also described for both food procurement and preparation. However, it was found to be secondary to money costs as a determining factor for procurement. Participants in the coastal areas talked about the effort to reach markets: “…*sometimes, when we feel we cannot walk, we just decide that there will be no vegetables on that day*”. (FGD 1; Female; 65 years_b) Apart from availability of local markets or locally grown produce, access to markets is an important factor and depends on the distance to be travelled and the availability of regular and affordable transport. When access becomes burdensome, the frequency of visits tends to drop. In difficult and hard-to-access areas, the nearest market was still too far away, which substantially increased the cost of food procurement. “*Our area is completely coastal. We have extremely salty sand, which prevents any vegetables from growing. So, all vegetables and fruits have to be purchased. There are also no local vegetable markets in our locality. If we have to buy any vegetables, we have to go to the [closest] city by bus*”. (FGD 1; Female; 65 years_a) In such areas, the travel expenditure was often higher than the price of the vegetables. When the balance was skewed with higher money and effort costs involved in the procurement of fruits and vegetables, the participants explained that they would limit their efforts to special occasions like festivals.

Some participants also talked about the time and effort involved in food preparation, which at times rendered the whole exercise pointless. Women working outside the home, had some extra money but often, no time: “*We don’t have much land. We do have a drumstick tree, so we get the leaves, but we don’t use it. It takes a lot of time to prepare that. I don’t have the time for that when I come back from work. I just try to make something fast for the children as they are hungry by then*”. (Interview 17; Female; 35 years)

*Category 2: Living within our means (Can we afford basic needs?)*

Households also had to ‘adjust’ or ‘cope’ with what is available in terms of financial resources. This type of coping or adjustment itself extracts a cost from those involved and is a factor in the food decision-making process. However, monetary costs were found to be the ‘rate-limiting’ factor in the process of food decision-making for low SES households.

Participants from the low SES, particularly in the first FGD, talked about ‘not spending what we don’t have’. “*Our children live within our financial situations, so their choices reflect this. Sometimes when we tell them that we cannot make something today, but will make it another day…they will listen to us*”. (FGD 2; Female; 39 years).

A household’s financial capability was often perceived in terms of the occupation of the various household members. “*We have no ‘sambathikam’ [financial resources]. I am a coir worker. I make what money I can from making coir. I get work only 5 or 6 days in a month. We cannot survive the rest of the month on that. This is the only work I know. I never studied… Look at my hands [blistered]. If I had any other way, I will not do this. …My mother is old and sick. She has pressure [hypertension] and sugar [type 2 diabetes mellitus] and she is not right in the head [mental illness]. She stays with me and I take care of her. My son sends me some money whenever he can, so we survive somehow*”. (Interview 11; Female; 58 years)

Access to financial resources however, has gendered implications. For women, lack of income leads to dependence on men for all expenses, loss of independence and negotiating power and inability to make decisions regarding what can be purchased, due to their lack of control over the purse strings. Women talked about the difficulty of having to approach their men to meet every need. As a result, the women’s own needs and those perceived as luxury, tend to be overlooked in favour of more high priority needs that cannot be ignored. “…. *Earlier, [when there were more manual jobs in road construction for women], if we wanted to buy something for our children or ourselves, we could do it. Now, we have to ask money for everything. Or, we have to ask them [men] to buy whatever we want, even if it is something like food*”. (FGD 1; Female; 66 years)

Women from the lower SES, had either no jobs or no regular income to keep the money coming in and this is a frustrating experience as described by one of the women: “*Most people complain that it takes a lot of time and effort to cut vegetables. That is only for those who don’t have time. Here, we have time, but no vegetables and no money to buy it*”. (FGD 2; Female; 67 years). In such cases, they had to rely on the amounts they received from their spouses to meet the food needs of the household. While the frequency of access to this amount varied, there was an observable pattern. In the middle SES and more affluent households, where the men had regular jobs, the women were often given a monthly allowance to take care of household and children’s needs, which mainly included food, and school expenses. In the lower SES, it was more erratic. Often, they got the money for food on a weekly basis depending on the number of days worked, while children’s school expenditure money was often given at the beginning of the term or borrowed by the women from their relatives. Women whose spouses worked in the Middle East, received money for longer periods, may be two to six months (depending on the spouse’s job) for household and children’s expenses; and also had greater autonomy in making day-to day decisions.

### Sub-theme 2: Finding the balance

The second sub-theme basically relates to how the benefits are balanced during the decision-making process.

*Category 1: Meeting household needs (Is it a household priority?)*

Meeting household needs was found to be a combination of meeting food-related needs, particularly basic needs; and also understanding and taking care of the differential needs of its members.

Meeting basic food needs was the main outcome of the food decision-making process. Even in the lower socio-economic strata, this was very evident, as satisfying hunger, which is the most basic function of food is somehow managed with whatever means was available. “*Most of the time, our meal would be only rice and fish. Since we are fish vendors, we have fish during the season…. We use chammanthi [dry coconut chutney] if we don’t have fish.*” (FGD 1; Female; 57 years) At this level, the nutritive value of foods or its effect on health, play no part in the decision process. When basic needs are perceived to be unmet, healthy food becomes a luxury that they cannot afford. “*How can we buy fruits and vegetables in our house? We will have to decrease something else. But, there is nothing to decrease. We use ration rice that costs two rupees. We won’t get anything else for that kind of price. We don’t get vegetables for less than thirty rupees now, because of the price rise. Shopkeepers refuse to give for less than thirty rupees. If we buy this pack in our house of seven members, I can only make one sambar and one aviyal and it will still not be enough for everyone. That is our situation*”. (FGD 2; Female; 67 years)

Household members also had differential needs, and it was important to understand these needs, which were prioritised and factored into the decision-making process. These were household priorities and often stemmed from the perceived needs, particularly for children, whose needs were prioritized in most households. Similarly, based on disease status, adult members of the household may have special food needs or restrictions. Households with a member with diabetes or hypertension usually had some routine in place to deal with food restrictions. Working men were also perceived to have special needs in terms of energy requirement, particularly those engaged in high intensity manual jobs, like farming or fishing. “*We are fishermen, we work through the night and most of the day. We need rest and good food…. Usually, we have only rice and fish and may be chammanthi. How will we work all day if we eat small amounts?*” (FGD 3; Male; 41 years)

*Category 2: Maximising household satisfaction (What is its value?)*

Household satisfaction seemed to be assessed based on the value of the procured or consumed foodstuffs, as perceived by different household members. Therefore, household satisfaction was maximised through the procurement of high-value foods.

Participants considered traditional values regarding food choices and practices and traditional beliefs about certain foods, like plantain and tapioca, as contributing to health: “*Our food habits are traditional…we don’t agree with any of these new habits like eating meat every day*”. (Interview 16; Female; 58 years) Similarly, regular food, mainly rice, and other traditional rice and legume-based preparations were considered healthy. Food habits that went against medical advice, health promotion messages or prevalent notions of healthy food were considered unhealthy. Certain foods like fruits were cold and could therefore not be consumed during most illnesses; while payar kanji (rice porridge with green gram and shallots) was ideal as it would both clean your body and provide enough nourishment.

Adulterated foods or foods believed to have a high probability of being adulterated or contaminated were considered unhealthy. This included ‘red’ rice, as well as fruits and vegetables. “*Most people use white rice now. Earlier, they used to use only red rice. First of all, red rice is expensive. On top of that, the red rice you get now is just coloured white rice. After one or two washes, all the colour comes off……Life is surrounded by adulterated things. We have to survive in the middle of these things*”. (FGD 3; Male; 57 years) The notion that they were paying to ingest harmful poisons was quite strong and many of the households mentioned pesticides in fruits as a reason for not buying fruits. “*They are full of chemicals. It is so poisonous, that we will get other diseases….*” (FGD 3; Male; 62 years). However, they also acknowledged that it was the higher price of fruits that promoted this unfavourable comparison. “*When you consider the price you have to pay for the chemicals in fruits, it is better not to eat it at all*”. (FGD 2; Female; 58 years).

Different foods were also attributed different values, by which they were prioritized. Some of the foods were considered regular fare and therefore needed no specific label to describe it. Along similar lines, ‘fruits’ were different types of small bananas. Most families grow a few banana trees in their own yard. They would purchase fruits like oranges and grapes, referred to as ‘other fruits’ from local markets only if they don’t get bananas.

Certain foods were however, attributed higher values based on the impression created by media advertisements, particularly those portrayed as healthy and necessary for growing children; often leading to higher than affordable food expenditure. Even among the low SES households, they would not hesitate to buy some of these more expensive foodstuffs at the cost of their regular food. “….*spend an average four-thousand rupees on food per month [household of seven members, including four children]… have to spend an extra one-to two-thousand on biscuits and powder items [health drinks] for small children in the house*”. (FGD 1; Female; 65 years_b)

The ability to buy also often imbued ‘good’ qualities to the food purchased, thus improving their attributed value. “*There are four working men in our home so, we can generally afford to buy good food*”. (Interview 14; Female; 23 years) The attributes of the job were also de facto transferred to the things they were able to afford. Government jobs were considered good jobs, because of the security they provide in terms of a regular income with old-age benefits: “*My son-in-law has a good job [government job] so, they buy only good food*”. (Interview 3; Female; 65 years). Consequently, what you got for free was often undervalued or even ignored: “*We had a big papaya tree in our yard. Only I used to eat the papaya from that. I eat a few pieces and then keep it in the fridge and then throw it away when it gets spoiled. This is what generally happens. Now it has been cut down*”. (Interview 16; Female; 58 years) Similarly, foods that one could not afford on a regular basis, like restaurant food, were often considered better than the food they could afford to buy and prepare at home.

*Category 3: Matching roles and expectations (Whose preference is more important?)*

Household expectations were tightly bound to the preferences and the role and position of its members in the household hierarchy; as opposed to household satisfaction described earlier that was linked to the value of the procured or consumed foodstuffs.

Habit, taste and comfort in routines were three attributes many of the participants described to explain the reason for certain preferences. “… *we have habit of seasoning with coconut oil and mustard. … it is unnecessary, but everyone likes the taste of that*”. (FGD 3; Male; 30 years); “*….fish is always fried in our house, it tastes better..*” (Interview 14; Female; 23 years); “In our house, we have to have tapioca everyday, except Sunday…”. (Interview 8; Female; 29 years) The importance given to these preferences and priorities often decided the way certain foods were cooked, based on positions and expected roles in the household. Preferences of husband and children (and sons-in-law when the daughter’s family was co-resident), were prioritized in most of the households. The preferences of the breadwinner of the house was also prioritized, partly because of a feeling of entitlement; and partly because of perceived needs, especially for men engaged in manual labour. Women often ignored their own preferences when weighed against the preferences of their family members. “*Only I eat that [vegetable dishes], so, I don’t feel like making it only for myself*”. (Interview 1; Female; 36 years)

When perceptions and notions of healthy food were in line with stated preferences of household members, practising healthy food habits became easier: “*….my husband prefers vegetables to meat, so we buy [vegetables] almost everyday*”. (Interview 9; Female; 26 years) Women talked about difficulties in this regard, particularly when there was a mismatch between the two: “*… we use lot of oil…difficult to reduce, he [husband] likes lot of fried things…*”(Interview 14; Female; 23 years); “*….we know that tapioca is not good for sugar, but in our house, it is compulsory*”. (Interview 8; Female; 29 years) This made it difficult in some cases, when foods were prepared to the preferences of one family member, usually the spouse, making other (s) unhappy (co-resident mother of the wife); leading to tensions in the family: “*If they [daughter and son-in-law] make food with too much salt, that day I only eat rice. I won’t take anything else*”. (Interview 3; Female; 65 years)

Both women and men considered food procurement and preparation as essentially a woman’s job. There was only one household where a man was routinely involved in these activities. In their roles as breadwinners, men would get access to luxury foods like snacks, for fruits like oranges and apples, which were not available in way-side markets, during special trips or when they receive their pay-packet.

Responsibility for food preparation lay exclusively with women. It imparts a sense of responsibility to the women to make the food tasty and palatable for the others: “*If we have to make something tasty, we need oil and salt. We know that we should decrease salt and oil but food won’t be tasty*”. (Interview 16; Female; 58 years); and a sense of entitlement and expectation to their spouse and grown-up children, particularly sons; that the food be prepared to their taste: “*If he [husband] doesn’t like something, he will get angry and he won’t eat….*” (Interview 1; Female; 36 years); “*When my son comes home, I make whatever he likes…*” (Interview 16; Female; 58 years)

### Main theme: Balancing expectations amidst limitations

The whole food decision-making process ran like a complex cost-benefit analysis where the costs of procuring or preparing foods (in terms of money, effort or time) were balanced within the boundaries set by the household needs, satisfaction and expectations. However, the burden of making adjustments and balancing this act within the means available at their disposal fell disproportionately on the women.

The central role of the household as opposed to the individual was also implied throughout the data. However, the woman of the household, who is the explicitly acknowledged decision-maker, carried out various activities based on available financial and other resources and the needs and preferences of the implicit decision-makers (most often, spouse and children).

### The food decision tree: understanding the household prioritization process

Based on the data and the analysis, we constructed a food decision tree to explain the process and gain further insight into the prioritization process. As the food decision-making was a complex process, it was also apparent that prioritizations take place at many levels. Thus the food decision-tree is a schematic representation of this process (Fig. 1), which usually starts with considering the purchase or procurement of specific foodstuffs, such as procuring greens from their yard. Based on our understanding, the prioritization process then involves five questions at different stages in the following order: 1) Is it the basic minimum they can afford to satisfy essential food needs? 2) Is it a household priority or preference? 3) What are the costs involved? 4) Whose preference? and 5) What is its value?

Firstly, if the household could not afford any other foodstuffs of their choice based on the resources available to them, then they would use it to satisfy their basic need. Secondly, if it were a priority, i.e. it was considered as essential based on the disease status or age of household members, particularly spouse and young children, the answer would again be affirmative. All other outcomes seemed to be cost dependent with the needs and preferences of spouse and children prioritised over everything else. Health as a consideration had a very low priority when looking at the procurement and consumption of fruits and vegetables or reduction in salt, sugar and oil and tended to be considered only when all other factors were favourable. It was often a by-product of healthier preferences of the more influential household members.

## Discussion

Food decision-making in the study setting had a larger household component than previously considered in the more high-income settings [36, 40]. In addition, we found that the most crucial decisional point was affordability in terms of money costs, followed by food preferences of husband and children. Moreover, household expectations were also tightly bound to the preferences of the household members. Hence it follows that practising healthy food habits became easier when perceptions and notions of healthy food were in line with stated preferences of household members. In terms of developing health interventions, the take-home message is the high priority given to the preferences of spouse and children and the sensory appeal of food. This is especially relevant when considering the low priority given to the health aspect of consuming fruits and vegetables or reducing salt, sugar and oil. It is likely to be a reflection of the routine decision-making, where health becomes an issue only when it is seriously compromised; rather than a specific deliberation regarding food choices or practices.

### Comparison with other decision-making models

In spite of the difference in the objectives of the respective studies and the methods employed (ethnographic vs. descriptive individual interviews and FGDs), four of the seven propositions outlined by Gillespie et al. have been confirmed in our model as well [32, 34], like their description of the process as an interactive balance of power, and food decisions as a reflection of a family’s values and goals [34]. The greater family or household component in their study is also a reflection of the many settings they have included. In addition, they also describe most food-related decisions as the result of routine or habitual behaviours; with situation-specific decisions being based on an assessment of priorities, alternatives and available resources [34], which was also true in our setting. Using poverty as a an example of a specific situation, they have further qualified well-thought decisions made in households as being within their value systems and hence, not disrupting community or family well-being and cohesiveness in any way [32]. This was partially true for our study as even low SES households made sure that basic food was provided with what was available. However, they went beyond what they could afford when it came to children’s needs. It is possibly a trade-off decision with the wellbeing of the prioritized members (children) taking precedence over the wellbeing of the adult members or even the financial wellbeing of the household. However, practising healthy food habits was easier when the perceptions regarding healthy food did not clash directly with the preferences of the household members, avoiding arguments and tension, thus increasing well being.

The food choice framework by The food choice framework differentiates between factors that influence what a person is able to buy and consume, specifically availability and monetary costs; and what a person chooses to buy and consume specifically, sensory appeal, familiarity, social interactions, personal ideology, media and advertising and health [47]. Time constraint was described as common to both sets of factors [47]. Most of these factors were identified in the present study as well. The effect of media and advertising were mentioned as affecting food decisions related to children but the data was too sparse to be included in the decision tree.

In other studies, health was a major factor affecting food choices in relation to fruits and vegetables [47] as well as in working mothers’ food choices [48]. In the present study, on the other hand, health was not a major decisional factor, which may be partially influenced by our focus on the five specific food components. Fruit and vegetable consumption was low in Kerala [12], and therefore probably not even factored into the health equation of most households. However, salt, sugar and oil are three components that were regarded by the participants as important contributors to the sensory appeal of the food, which was found to be an important decisional factor. Many of the quotations point towards reluctance on the part of the women to sacrifice the taste and flavour of food, which may make it unpalatable for their spouse or children; and a general unwillingness on the part of the men to consume food that is not to their taste. Both these circumstances contribute to our finding that health was not a major consideration for food choices concerning these five components. A similar disconnect between diet and health was found in another study looking at factors affecting food choices of low-income women [49]. As our participants were predominantly from low- and middle-income households, it is possible that a study among more affluent households may show different results in this regard [49].

Studies looking at consumer decision-making, have found a greater role and autonomy for women in decision-making related to food [28]. However, both our study and those done elsewhere have found that while women may be responsible for making food-related decisions, they adjusted their decisions based on spouse’s and children’s preferences, thus reducing conflict in households [29]. Children’s preferences however tend to be prioritised differently in different cultures. In emerging economies, with low total fertility rate (like Kerala state in India), children’s needs tend to get prioritised [50]. In contrast, in contexts where children are seen in terms of an extra hand to contribute to the family economy, the gender and age of the child will determine whether his or her needs are prioritised [27, 51].

We found that food decision-making was mainly related to procurement and preparation, and that was why we concentrated on these aspects, and food procurement in particular, for the decision tree. The food choices of individual members in a household were therefore limited to what had been procured and prepared for the consumption of all the members. Moreover, choice or bargaining power was dependent on the individual’s position in the household hierarchy [27]. The responsibilities related to procurement and preparation, were generally carried out by a few members on behalf of the whole household, with the process being influenced by the preferences and needs of the other members [34]. Even decisions related to food preparation had to start with decisions to procure the commodities needed to make the particular preparations. This was the reason why we chose to initially focus on female heads of the households.

We have in line with Lancaster found that household decision-making cannot be equated to individual decision-making [52]. When we describe a household-level decision process, it implies more than a household arbitrarily carrying out the decisions made by its most-influential or dominant member. Instead, the decision-making is a process, comprising a series of tacit negotiations, influenced by the explicit needs and the unspoken preferences of all the household members, and where the decisions are the end result. This understanding is essential while designing dietary interventions in this and similar settings as an intervention designed for and targeting individuals is unlikely to have substantial impact.

### Power and gender dimensions of the household decision-making process

The household food decision-making process was all about power relationships within families and in the communities, which has gendered implications. In the present study setting, only one of the women participants had formal employment, hence it was not surprising that the power within these households rested with their breadwinning spouses. This is not a new finding and is part of the established understanding related to human societies that greater decision-making power within households rests with the members contributing the greater share of the household income [53].

Husbands’ and children’s needs and preferences seem to be prioritised in most settings, including our own. Studies conducted in Belgium and the United States, with vastly different cultures and norms as compared to our study setting, have shown similar results [33, 34]. Women have been seen to adopt a more community or household oriented approach to money management and decision-making, while men take a more individualistic approach [29]. Gender role expectations, which are socially determined [27], underlay both the primary responsibility and the prioritising [8] i.e., a ‘good home-maker’ was supposed to be able to do this, just as she was expected to balance the expenses within the available resources. The same roles and expectations also dictated what each member was entitled to. The husband and older sons on the one hand were entitled to have their preferences prioritised; their favourite foods cooked to their taste. Women on the other hand, did not eat or prepare many of the foods (even healthier choices than their spouse’s), because they did not feel entitled to be able to spend their time and effort on personal preferences. Norms or perception of norms, thus “set the limits to bargaining” [27], p. 15 by limiting bargaining power and options.

### Implications of the findings

Here we describe implications both for policies, and design and development of intervention studies.

1. An intervention aimed at promoting a facilitating environment for behaviour change within the household; and any tools and materials prepared, should be applicable at the household level.
2. Women can serve as good proxies to represent the household in food-related matters, as they are the explicit decision-makers in the household, and as they have good knowledge about the limitations imposed by their family circumstances as well as those indicated by the implicit decision-makers (spouse and children).
3. Specific strategies should be evolved to engage both men and children actively in the household behaviour change process.
4. Low SES households need specific policy measures, particularly targeted food subsidies, if they are to have a comparable chance of success as other SES groups.
5. It is important, both in intervention studies and mass media campaigns, to address the lack of or limited awareness of the link between diet and NCDs, the risk of NCDs for individual family members, and the benefits of simple prevention measures.
6. The role of mass media advertisements in promoting children’s foods is an area that requires careful evaluation. It is important for the content of food-related advertisements to be carefully regulated, with the actual food value spelt out for special products, especially those for children. Packages should carry specific warning on their status as food supplements; and advertisers should not be allowed to promote these foods as having more value than or as substitute for regular diet.
7. Introduction of local markets in difficult geographic locations, or improving cost-effective transportation means; either for people to access markets or for foodstuffs to be brought to the people, could dramatically change behavioural outcomes within very short time periods.

### Limitations

First, the low male participation in the individual interviews was perceived as a limitation during the study and the third FGD was organized with a view to address this. It was however, joined by four women, which could have led to a dilution of the views that the men may have otherwise expressed. It is equally possible that the views of the women prompted discussion on topics, which may not have come up in an exclusive men’s group. However we decided to retain this FGD data for analysis for the following reason. Initially two focus groups were conducted among women and it became clear that we needed to listen to the men and especially to understand the stated and unstated preferences that women based their food decisions on. Hence, we decided to conduct in-depth interviews in households and invite both the female head of the household along with another member. However, we soon realized that, except in one household, men were reluctant to be interviewed on a subject they considered the women’s domain. We had used women volunteers from the community as gatekeepers to gain access and invite the participants. Instead of individual interviews, we arranged a focus group with men, which finally included a few women as described above. This focus group discussion clarified many of the issues brought up by the women previously, regarding the importance of taste and thereby men’s and children’s implicit roles in decision-making. Since we felt that the contributions that came out of the men’s focus group discussion were important and supported many of the statements made by women, we decided to retain this data in this analysis.

Second, as the participants in the study were mostly women, the expectations described are their perceived expectations based on stated preferences and past behaviour to prepared food from other members of the household. The focus group conducted among men also supported these expectations. We did not interview children, however many of the participants described the reactions of their children to certain foods, based on which they would decide whether or not to prepare the food item again.

## Conclusions

The food decision-making process occurred at the household level. While women were the explicit decision makers, the process was influenced by the preferences of spouse and children, as well as cost considerations. Children’s needs were highly prioritized; with foods perceived to be important for children being procured in spite of their high cost. Food procurement and preparation were universally considered a woman’s role and responsibility; and women disproportionately bore the burden of meeting the household’s needs and fulfilling other members’ expectations within the available resources. The socio-economic status of households was an important factor that limited or enhanced the women’s ability to manoeuvre the fine balance between managing costs and meeting needs and expectations. The findings have major policy implications, regarding the need for targeted food subsidies for low SES households; regulation of media advertisements of children’s foods; and raising awareness about the link between food and non-communicable diseases, which could be a focus in mass media campaigns on prevention.

## Additional files

Additional file 1:
**Guidelines: FGDs & In-depth Interviews.**
Additional file 2:
**Example of meaning units and abstraction till sub-themes for two data sections (different colours) from one participant in FGD 1.**
