# Supplementary material for: Balancing expectations amidst limitations: the dynamics of food decision-making in rural Kerala
Source: BMC Public Health. 2015 Jul 12;15:644. doi: 10.1186/s12889-015-1880-5 (PMC4499445; doi:10.1186/s12889-015-1880-5)
Supplement: Additional file 2: — Example of meaning units and abstraction till sub-themes for two data sections (different colours) from one participant in FGD 1. [file 12889_2015_1880_MOESM2_ESM.pdf]

Additional file 2

Example of meaning units and abstraction till sub-themes for two data sections (different colours) from one participant in FGD 1

<table>
<tr><th>Meaning units</th><th>Condensed meaning units</th><th>Codes</th><th>Sub-categories</th><th>Categories</th><th>Sub-themes</th></tr>
<tr><td>To get a pack of vegetables that cost minimum 20/-, we have to pay a bus fare of 10/-. So, we need total 30/-. Some of us have to take an auto for 45/- to get this 20/- vegetables.</td><td>Transport is more expensive than the vegetables</td><td>Expensive travel</td><td>Financial access to market - Money costs</td><td rowspan="5">Monetary and other costs</td><td rowspan="9">Counting and meeting the costs</td></tr>
<tr><td>Mornings often there is no bus.</td><td>No transport to market</td><td>Lack of transport to market</td><td rowspan="2">Physical access to market – Money and effort costs</td></tr>
<tr><td>For return, if the auto driver is nice, he may charge only 30/-. Or else we have to walk.</td><td>Market is too far away – too much to spend</td><td>Market is too far away</td></tr>
<tr><td>If we walk in this sun, we will be so tired or sick with cough or breathing difficulty that next day we can't go anywhere, do anything.</td><td>Market is too far away – too much effort</td><td>Cannot cope the next day</td><td rowspan="2">Adjustment or coping costs</td></tr>
<tr><td>Sometimes, when we feel we cannot walk, we just decide that there will be no vegetables on that day.</td><td>It is too much trouble to get vegetables</td><td>Adjust with no vegetables</td></tr>
<tr><td>We use 'chammanthi' if we don't have fish.</td><td>Use only coconut chutney and rice</td><td>Manage basic needs</td><td rowspan="2">Manage with what we have</td><td rowspan="4">Living within our means</td></tr>
<tr><td>Most of the time we don't use vegetables.</td><td>Don't use vegetables regularly</td><td>Cannot afford vegetables regularly</td></tr>
<tr><td>We don't have money of our own so, we can't do anything.</td><td>No money of our own</td><td>Women have no money of their own</td><td rowspan="2">Financial resources and dependence</td></tr>
<tr><td>We can only use the money that our husbands give us.</td><td>Money given by spouse</td><td>Money has to be given by spouse</td></tr>
</table>
